# Assessment and Discussion of the Steady-State Determination in Zeolite Composite Membranes for Multi-Component Diffusion

**DOI:** 10.3390/membranes15100301

**Published:** 2025-10-02

**Authors:** Katarzyna Bizon, Dominika Boroń, Bolesław Tabiś

**Affiliations:** Faculty of Chemical Engineering and Technology, Cracow University of Technology, 31-155 Kraków, Poland; dominika.boron@pk.edu.pl (D.B.); boleslaw.tabis@pk.edu.pl (B.T.)

**Keywords:** zeolite, composite membranes, multicomponent diffusion, steady states, separation, algorithms

## Abstract

A versatile, clear, and accurate method for determining the steady states of multi-component diffusion through composite membranes is presented in this study. This method can be used for simulating and designing membranes with any support orientation with respect to the zeolite film. In the mathematical model of the membrane, it was assumed that mass transport in the zeolite layer occurs by surface diffusion in accordance with the generalized Maxwell–Stefan model. Diffusion in the macroporous support was described by the dusty gas model (DGM). An alternative model of diffusion in the zeolite was proposed to the universally accepted model, which uses a matrix of thermodynamic factors **Γ**. Thus, the difficulty of analytically determining this matrix for more complex adsorption equilibria was eliminated. This article is dedicated to methodological and cognitive aspects. The practical features of the method are illustrated using two gas mixtures as examples, namely {H_2_, CO_2_} and {H_2_, *n*-C_4_H_10_}. The roles of zeolite and support in the separation of these mixtures are discussed. It was demonstrated under what circumstances the presence of the support can be neglected in the steady-state analysis of the membrane. The effect of the alternative application of the dusty gas model or viscous flow only in the microporous support was discussed.

## 1. Introduction

This paper was inspired by several papers published in the first decade of the 21st century on the modeling of multi-component diffusion in composite zeolite membranes. We quote the opinions expressed in these articles concerning how to determine steady states in such objects. In our opinion, these opinions are questionable or even misleading. Therefore, it is difficult to state unequivocally whether the results obtained from the methods discussed in the mentioned articles are reliable. Consequently, we present our own method for determining steady states for multi-component diffusion in composite microporous membranes, particularly with the use of zeolite films.

The proper determination of the steady state is the foundation for designing separation processes in membrane apparatuses, which generally operate under steady-state conditions. Additionally, it serves as a tool for verifying the dynamic models of such equipment. This is because the steady state represents the asymptotic solution of such models that corresponds to the time of the process tending to infinity.

The issue itself is crucial due to the significant importance of separating mixtures using membranes. Moreover, in recent years, there has been a substantial increase in experimental works on the preparation and production technology of zeolite composite membranes [1,2]. The novel technologies are modifications of the two basic methods for obtaining composite membranes, namely, the in situ and ex situ (second growth) methods. To a great extent, recent studies have focused on modifying zeolite membranes, with the aim of, among other things, reducing pore size [3] or functionalizing their surface [4]. The improvement in techniques for producing membranes is due to their application in many important industrial processes today, i.e., biogas upgrading [5,6], natural gas purification [7,8], CO_2_ capture [9,10], or hydrogen production [11]. An example of the effective use of membrane techniques is the separation of the {H_2_, CO_2_} mixture. Traditional carbon dioxide separation processes most often use cryogenic distillation, anion absorption, or pressure swing adsorption (PSA) technology [12,13]. Despite the high efficiency of these methods in obtaining high-purity hydrogen, their use is associated with high energy consumption and process complexity. In recent years, several main types of membranes used for the separation of hydrogen and carbon dioxide have been distinguished in the literature, such as zeolite membranes [14], polymer membranes [15], metals and metallic membranes [16,17], and various types of composite membranes [18]. Compared to polymer membranes, zeolites offer higher selectivity and stability at high temperatures, although often at the expense of lower permeability [15]. On the other hand, metallic membranes, especially palladium membranes, exhibit almost perfect H_2_ selectivity, but are expensive and sensitive to contamination [16,17]. Therefore, zeolite membranes offer a favorable compromise between separation efficiency, stability, and chemical resistance, making them competitive with conventional technologies and other types of membranes, especially in harsh or high-temperature conditions.

It is somewhat surprising that, despite numerous and advanced studies on the synthesis and technology of composite membranes, insufficient attention has been paid to methods for determining the steady-state conditions of multi-component diffusion in such objects. In a paper authored by van de Graaf et al. [19], it is stated that until 1999, “no attempts have been made to describe the separation performance of zeolite membranes quantitatively.” This implies that theoretical works in this subject area date only from the 21st century.

Initially, the properties of microporous membranes without supports were modeled and analyzed theoretically. As representative, the works of Krishna and co-authors [20,21,22,23,24,25,26] and Kapteijn and co-authors [27,28,29] can be mentioned. These works focused on the specification of membrane selectivity, the determination of molar flux densities of individual mixture components, or the estimation of diffusion coefficients. However, all these studies concerned single zeolite layers, known as bare membranes. Thus, they did not concern composite membranes.

In the cited paper by van de Graaf et al. [19], it was stated that the support layer cannot be neglected. A laconic statement was made that the steady state of the membrane was determined by solving the equations of dynamics for a sufficiently long diffusion time. There is no information regarding the basis on which the arrival at this steady state was determined, and what quantity was considered to be sufficiently invariant. Nevertheless, it can be considered to be a work of great theoretical importance.

In the course of time, studies indicating the role of resistance to mass transport in the supports started to appear. Some examples are the publications of Farooq and Karimi [30], Bruijn et al. [31], Hanebuth et al. [32], Wirawan et al. [33], or the publication of Kangas and co-authors [34] on the theoretical and experimental analysis of steady states in a composite membrane for the separation of H_2_ and CO_2_.

Nowadays, a composite membrane is usually defined as a barrier with at least two layers, consisting of a permselective nanoporous film and a meso- or macroporous support. In order to justify undertaking our own research on the correct determination of steady-state conditions in such membranes, we first quote some opinions provided in selected articles published already in the 21st century.

The work of Skoulidas and Sholl [35] reports the results of numerical simulations of methane diffusion through a silicate membrane with a porous support in the presence of helium as sweep gas. It was assumed that helium is also adsorbed in zeolite. The method of lines was used to determine the steady states. It is a numerical method used to solve partial differential equations [36]. Thus, the equations of dynamics were integrated until the steady state was reached. Again, the criterion for obtaining such a limit state was not provided in this work. Instead, an alarming statement was provided that the equations describing mass transport in the zeolite layer and in the support were integrated independently. In contrast, the equations of the entire membrane model were coupled only by the boundary conditions at the interface between the two layers. According to the authors, such a statement, in light of the mathematical formalism, is incorrect. It follows from the way the membrane as a whole works that the concentration distributions of the diffusing components in the zeolite layer depend on the concentration distributions in the support, and vice versa. In addition, the distributions of concentrations in the zeolite and in the support depend on the boundary conditions at both ends of the membrane as a whole. From this mathematical formalism, it is concluded that for numerical simulations of the dynamics of composite membranes, it is necessary to know the concentration distributions of the components in both the zeolite and the support at each time instant *t*. The concentrations of the components can be expressed in various equivalent forms, e.g., by fractional occupancies or equilibrium partial pressures. With the above considerations in mind, it is difficult to say unequivocally whether the results reported in the aforementioned paper actually refer to steady states.

The method of lines was also used to determine steady states in the work of Wirawan and co-authors [33]. A combination of different mass transport mechanisms during the diffusion of a {H_2_, CO_2_} mixture through a composite membrane incorporating a permselective silicalite-1 layer deposited on a double support was reported. The authors used a discretization of the diffusion equations in zeolite into 50 subintervals. Molar flux densities through the supports were calculated using analytical relationships determined for single, pure components. Such expressions, however, do not take into account molecular diffusion. This reduces the model’s generality. In addition, they were obtained for a steady state. For this reason, they are not compatible with the dynamic model of the zeolite film. The authors proposed an iterative optimization method for determining partial pressures at the zeolite–support interface. This resulted in increased calculation time and required the specification of a criterion for the accuracy of calculations. The primary purpose of the work under consideration was to determine the missing values of the model parameters. An additional external iterative loop was required for this purpose. However, in the case of adopting an incorrect model, the fitted parameters were also determined incorrectly. Taking the above into account, the membrane model and the method of determining steady states, as presented in the work in question, do not guarantee reliability.

A somewhat limited and brief discussion of the effect of support on the dynamics of a zeolite composite membrane was given by Martinek et al. [37]. The authors used the finite difference method to solve the equations of membrane dynamics. Unfortunately, the number of required grid nodes was not specified, either with respect to the spatial variable or the time coordinate *t*. The diffusion time required to reach a steady state was also not evaluated. Instead, the discussed paper provides a statement that may be misleading. The authors write that if “the permeate partial pressures were not known, the model was run iteratively to determine them for known total permeate pressures” [37]. Such a statement is correct only for single-component diffusion. In the case of two- or multi-component diffusion, it is false, since a given total pressure can correspond to an infinite number of partial pressures of the individual components, resulting in a specified total pressure.

Hanebuth et al. [32] analyzed the steady state of a zeolite composite membrane for the diffusion of a binary mixture {H_2_, SF_6_}. However, the authors did not account for hydrogen adsorption in the zeolite, and its diffusion was described by the Knudsen model. This means that only single-component surface diffusion occurs in zeolite. On the other hand, mass transport in the support was described by the dusty gas model. No information was given on how the mass balance equations in both layers were integrated. The algorithm for determining steady states requires iterative calculation of partial pressures at the zeolite-support interface, similar to the study by Wirawan et al. [33]. It does not emerge from the material in the discussed article how to determine the steady state of a composite membrane for multi-component diffusion, i.e., for a process in which surface diffusion of more than one component occurs in a zeolite film.

Recently, Kangas and co-authors [34] proposed the determination of the steady states of a composite membrane for multi-component diffusion based on a steady-state model. As is well known, in such models, the time variable *t* does not occur. Therefore, there is no need to determine the diffusion time required to reach the steady state. From the mathematical formalism’s perspective, this approach is valid. However, it turns out that to determine the steady state, the authors initially used a finite-dimensional approximation involving a discretization of the model equations with respect to the spatial variable. As a result, a large set of nonlinear algebraic equations was obtained. This is a significant drawback of such a method. Indeed, to determine the steady state, it is necessary to determine the approximate values of a large number of variables. This is because their number is proportional to the approximation grid employed, to the number of layers in the composite membrane, and to the number of diffusing components.

In view of the aforementioned gaps and inconsistencies, the development of a comprehensive, clear, and accurate method for determining the steady state of multi-component diffusion across composite membranes was proposed and adopted as the primary goal of the work. As an illustration of the method, an additional objective was to determine the concentration distributions in the different layers of such membranes. The overall efficiency of the membrane is determined by these profiles. The importance of quantitatively analyzing concentration distributions in microporous adsorbents was highlighted by some researchers in the 1990s [38] and in more recent publications [39]. The method proposed in the study can be utilized for simulating and designing membranes with any orientation of the support with respect to the zeolite layer and with different numbers of diffusion layers. Additionally, the method applies to any number of diffusing components. In such cases, the idea of the procedure is maintained.

## 2. Mathematical Model and Numerical Procedures

### 2.1. Idea of the Method

The purpose of the following paragraphs is to discuss the method for the determination of the steady state states of composite membranes. A well-known and straightforward shooting algorithm was utilized. The number of unknowns that are sought in the external Newton algorithm is small and equal to the number of diffusing components *K*. The internal algorithm is used to integrate systems of ordinary differential equations describing steady states in the zeolite and in the support. The membrane model and the method for determining steady states can be extended by taking into account the diffusive mass transport of non-adsorbable gases in the pores of the zeolite. Such gases can be negligibly poorly adsorbable components of the mixture or purposely used sweep gases.

To formulate the mathematical model of the membrane, it was assumed that mass transport in the zeolite layer occurs via surface diffusion according to the generalized Maxwell–Stefan (M-S) model [20,22,27,29]. Diffusion in the support was described by the dusty gas model (DGM) [32,33,39,40].

At the core of the generalized Maxwell–Stefan model lies the assumption of thermodynamic equilibrium between the solid and the gas phase. This implies equality of chemical potentials of the components, i.e., $\mu_{i}^{z}=\mu_{i}^{g},(i=1,\ldots ,K)$. Thus, at each site of the zeolite, the following relationships are valid:

(1) $$\mu_{i}^{z}=\mu_{pi}^{o}+RT\ln\left(\frac{p_{i}^{z}}{p^{o}}\right)$$

from which

(2) $$\frac{d\mu_{i}^{z}}{dz}=\frac{d\mu_{i}^{g}}{dz}=\frac{RT}{p_{i}^{z}}\frac{dp_{i}^{z}}{dz},\;p_{i}^{z}=p_{i}^{z}(q_{1},q_{2},\ldots ,q_{K});\;(i=1,2,\ldots ,K)$$

where *K* is the number of diffusing components.

It follows from Equation (1) that to model diffusion in zeolite, one can alternatively use as state variables concentrations *q_i_* expressed in moles per kg of zeolite, fractional occupancies θ*_i_*, or equilibrium partial pressures *p_i_^z^*. The third approach is used in the present work. It turns out that this approach eliminates the need to calculate a matrix of thermodynamic factors **Γ**, which is commonly adopted in theoretical works. Sorption isotherms are generally given in the literature as functions *q_i_* = *q_i_*(*p*_1_, *p*_2_, …, *p_K_*). Meanwhile, for the analytical determination of the matrix **Γ**, the inverse functions are required, i.e., *p_i_* = *p_i_*(*q*_1_, *q*_2_, …, *q_K_*), since

(3) $$\Gamma_{ij}=\frac{\theta_{i}}{p_{i}^{z}}\frac{\partial p_{i}^{z}}{\partial \theta_{j}},\;\theta_{i}=\frac{q_{i}}{q_{i}^{*}},\;(i,j=1,\;2,\ldots ,K)$$

Unfortunately, the analytical determination of these inverse functions is feasible only for a limited number of the simplest isotherms, which generally makes simulations and process calculations difficult [41,42]. In an earlier publication by the authors from 2024 [43], the correctness of an alternative model was demonstrated, i.e., one that employs equilibrium partial pressures instead of fractional surface occupancy to model the dynamics of bare zeolite membranes.

In order to explain the meaning of the previously mentioned diffusion time required to reach steady states, the results of the dynamic analysis of a bare membrane, i.e., without support, are presented below. As is well known, the presence of support reduces the molar flux densities of the diffusing components, thereby increasing the diffusion time. This means that the analysis of the dynamics of the bare membrane can be used to determine the limiting, i.e., the shortest time required to reach the steady state. The use of the support increases this time.

When the partial pressures are taken as state variables, the dynamic model of such a bare membrane is given by a system of partial differential equations:

(4) $$L^{2}\frac{\partial p^{z}}{\partial t}=J^{-1}\frac{\partial }{\partial \xi }\left\{{\left[B^{z}\right]}^{-1}\Delta \frac{\partial p^{z}}{\partial \xi }\right\},\;J=\frac{dq}{dp^{z}}$$

where ξ is the dimensionless spatial coordinate in the zeolite, while **J** is the Jacobi matrix of the partial derivatives of the concentrations in the zeolite phase *q_i_* with respect to the partial pressures *p_i_^z^*.

The matrices **B***^z^* and **Δ** take the following form:

(5) $$B^{z}={\left[\begin{array}{cc} \sum_{k=1,k\neq i}^{K}\frac{q_{k}}{q_{k}^{*}D_{ik}^{z}}+\frac{1}{D_{i}^{z}}; & \left(i=j\right)\\ -\frac{q_{i}}{q_{j}^{*}D_{ij}^{z}}; & \left(i\neq j\right) \end{array}\right]}_{K\times K}$$

(6) $$\Delta =\left\{\begin{array}{cc} \frac{q_{i}}{p_{i}^{z}} & \left(i=j\right)\\ 0 & \left(i\neq j\right) \end{array}\right.$$

**Figure 1:**
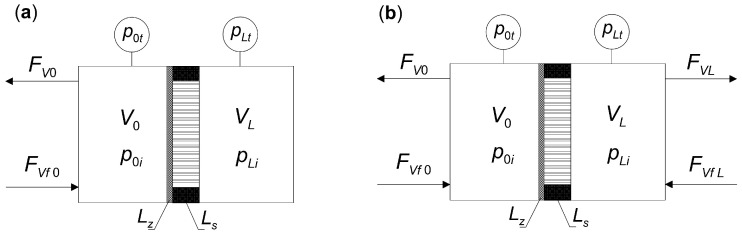
(**a**) A semi-closed Wicke–Kallenbach diffusion cell for studying the diffusion dynamics in composite membranes. (**b**) A two-sided opened Wicke–Kallenbach diffusion cell for studying the steady state in zeolite composite membranes. The figure shows a zeolite membrane of thickness *L_z_* with a support of thickness *L_s_*.

A classic and well-established method for experimentally studying diffusion dynamics in bare and composite membranes is the use of a semi-closed Wicke–Kallenbach (W–K) diffusion cell, as illustrated in Figure 1a. Meanwhile, Figure 1b shows a two-sided open diffusion cell, in which the processes are usually carried out under steady-state conditions.

**Figure 2:**
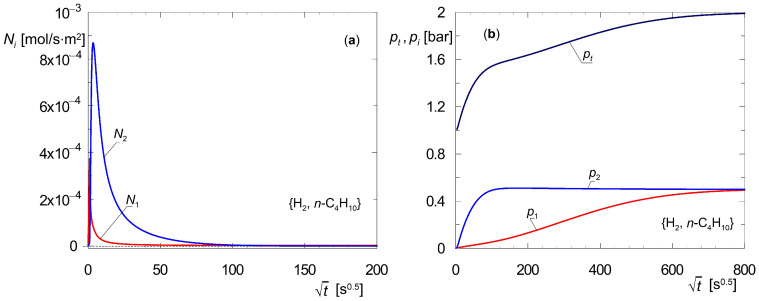
Dynamic characteristics of transient diffusion of mixture {A_1_, A_2_} = {H_2_, *n*-C_4_H_10_} across silicate membrane with *L* = 4 × 10^−5^ m for *y*_1_(0, *t*) = *y*_2_(0, *t*) = 0.5; *y*_1_(1, 0) = *y*_2_(1, 0) = 0.001; *p_t_*(0, 0) = *p_t_*(1, 0) = 1 bar; (**a**) time trajectories of molar flux densities *N_i_*; (**b**) time trajectories of partial pressures *p_i_* and total pressure *p_t_* in the closed volume *V_L_* of W–K cell (parameter values based on Krishna and Paschek [21]).

A system of differential Equation (4) was solved using the method of lines [36]. Figure 2 illustrates the time trajectories of two measurable quantities, i.e., molar flux density and total pressure. The partial pressures of the components are also provided. All these quantities refer to the closed volume *V_L_* (Figure 1a) and for a bare membrane, for which *L_s_* = 0. The values of the model parameters were adopted from available literature data [21]. The method for determining these trajectories was discussed in detail in an earlier publication [43].

Figure 2 shows the dynamic characteristics of the membrane that contains time trajectories for the diffusion of the binary mixture {A_1_, A_2_} = {H_2_, *n*-C_4_H_10_} in accordance with the data reported in the work of Krishna and Paschek [21]. The time trajectories of molar flux densities *N_i_*, partial pressures *p_i_*, and total pressure *p_t_* were derived from simulations of the transient diffusion of a strongly adsorbable component, i.e., *n*-butane, in the presence of hydrogen as sweep gas.

The task is to evaluate the time required to reach the steady state. Let the quantities used for this purpose be the time trajectories of the molar flux densities *N_i_*. Such a procedure is often used in the literature. Examples can be found in the work of Kapteijn et al. [28], van de Graaf et al. [19], Krishna and Paschek [21], or Krishna and Baur [23]. Figure 2 shows that individual variables, i.e., *N_i_*(*t*) and *p_i_*(*t*), change at different rates. Furthermore, it takes longer for partial pressures to settle than for molar flux densities. Therefore, partial pressures *p_i_* should be used to determine the time required to reach a steady state, rather than molar flux densities *N_i_*.

The measure of reaching a steady state is the settlement of the partial pressure *p*_1_, as it tends toward this state more slowly than *p*_2_. In laboratory tests, e.g., using a Wicke–Kallenbach chamber, partial pressures are not measured. Instead, the total pressure *p_t_* is measured. Figure 2b shows that the function *p_t_*(*t*) is a good measure of the time needed to reach the steady state. Therefore, choosing the appropriate variable and tracing its trajectory is a fundamental matter.

The analysis of the dynamics of diffusion across a bare zeolite membrane gives rise to two practical conclusions:

(a) During dynamic simulations of diffusion in zeolite membranes, changes in all state variables should be tracked, and the magnitude that varies in the slowest manner should be selected to evaluate the arrival at the steady state; moreover, such information should be included in the published scientific report;
(b) The use of partial pressures in the mathematical model simplifies such an assessment, as it indicates that partial pressures tend to approach the steady state in the slowest way, and thus also the measured total pressure.

### 2.2. Determining the Steady States of Multi-Component Diffusion in a Composite Membrane

Let us assume that the composite membrane consists of a single layer of zeolite and a single layer of support. The support can be located on either the retentate side or the permeate side, as shown in Figure 1. Steady-state operating conditions for the membrane may exist only when the volume *V_L_* is also open to the surroundings, as it is *V*_0_ (Figure 1b). Such a situation is encountered in industrial membrane apparatuses for the separation of gaseous mixtures. Then, for the process of multi-component diffusion through a flat composite membrane, the mass balance equations in the zeolite and support layers take, respectively, the form

(7) $$\frac{dp^{z}}{d\xi }=-\frac{L_{z}}{(1-\varepsilon_{z})\rho_{z}}\Delta^{-1}B^{z}N^{z},\;(p^{z}={\left[p_{1}^{z},p_{2}^{z},\ldots ,p_{K}^{z}\right]}^{T})$$

(8) $$\frac{dp^{s}}{d\zeta }=-L_{s}RT\cdot B^{p}\cdot N^{s}-\frac{B^{e}}{\eta }\frac{dp_{t}}{d\zeta }\cdot E\cdot p^{s},\;(p^{s}={\left[p_{1}^{s},p_{2}^{s},\ldots ,p_{K}^{s}\right]}^{T})$$

where ξ and ζ are dimensionless coordinates in the zeolite and support, respectively, defined as

(9) $$\xi =\frac{z}{L_{z}}\in [0,1],\;\zeta =\frac{x}{L_{s}}\in [0,1],\;z\in [0,L_{z}],\;x\in [0,L_{s}]$$

The total thickness of the entire composite membrane, however, is *L* = *L_z_* + *L_s_*. The equilibrium local partial pressures in the zeolite are contained in the vector **p***_z_*. The vector **p***_s_* represents the set of partial pressures in the support. The vectors **N***_z_* and **N***_s_* contain the molar flux densities. For flat membranes and steady-state conditions, the following equation holds:

(10) $$N^{z}=N^{s}=N$$

The matrices **B**^z^ and **Δ** are defined beforehand (Formulas (5) and (6)). The inverse of the diagonal matrix **Δ** is calculated analytically based on the assumed thermodynamic equilibrium model *q_i_* = *f_i_*(*p*_1_, *p*_2_, …, *p_K_*), (*i* = 1, 2, …, *K*). The matrices **B***^p^* and **E** contain elements as follows:

(11) $$B^{p}={\left[\begin{array}{cc} \sum_{\begin{array}{l} j=1\\ j\neq i \end{array}}^{K}\frac{y_{j}}{D_{ij}^{e}}+\frac{1}{D_{Kn,i}^{e}}; & \left(i=j\right)\\ -\frac{y_{i}}{D_{ij}^{e}}; & \;(i\neq j) \end{array}\right]}_{K\times K},\;\left(y_{i}=\frac{p_{i}}{p_{t}}\right)$$

(12) $$E={\left[\begin{array}{cccc} \frac{1}{D_{Kn,1}^{e}} & 0 & ..... & 0\\ 0 & \frac{1}{D_{Kn,2}^{e}} & ..... & 0\\ : & : & : & :\\ 0 & 0 & 0 & \frac{1}{D_{Kn,K}^{e}} \end{array}\right]}_{K\times K}$$

while

(13) $$D_{Kn,i}^{e}=\frac{4}{3}K_{0}\sqrt{\frac{8RT}{\pi M_{i}}},\;K_{0}=\frac{\varepsilon_{s}}{\tau }\frac{d_{p}}{4}$$

and

(14) $$B^{e}=\frac{\varepsilon_{s}}{\tau }\frac{d_{p}^{2}}{32}$$

The results of the numerical simulations shown below in the subsequent figures were obtained assuming that the diffusive exchange coefficients in matrix (5) were calculated according to the following relationship [44,45]:

(15) $$\ln D_{ij}^{z}(\theta )=\frac{\theta_{i}}{\theta_{i}+\theta_{j}}\ln D_{i}^{z}(\theta )+\frac{\theta_{j}}{\theta_{i}+\theta_{j}}\ln D_{j}^{z}(\theta )$$

As suggested in the literature [21,23], it was assumed that the diffusion coefficients of the individual components depend linearly on the fractional occupancies

(16) $$D_{i}^{z}(\theta )=D_{i}^{z}(0)\cdot (1-\theta_{1}-\theta_{2}-\ldots -\theta_{K}),\;(i=1,2,\ldots ,K)$$

where

(17) $$D_{i}^{z}(0)=\underset{\theta \to 0}{\lim}D_{i}^{z}(\theta )$$

Formula (16) has the advantage that it does not require the knowledge of additional parameters, as in the nonlinear model proposed by Reed and Erlich [46].

Since the membrane operates under steady-state conditions, there is no mass accumulation at any location within the membrane. Also, it does not occur at the zeolite–support interface. This implies the equality of partial pressures at this interface:

(18) $${\left.p^{z}\right|}_{\xi =1}={\left.p^{s}\right|}_{\zeta =0}$$

Two membrane configurations are analyzed:

(a) Support located on the permeate side, as in Figure 1;
(b) Support located on the retentate side.

The form of boundary conditions for the entire composite membrane depends on the orientation of the support with respect to the zeolite. Generally, they are given in the form of so-called boundary conditions of the first type (i.e., Dirichlet boundary conditions). For a support located on the permeate side, we have

(19) $$p^{z}=\varphi ,\;(\xi =0)$$

(20) $$p^{s}=\psi ,\;(\zeta =1)$$

If the support is located on the retentate side, then

(21) $$p^{s}=\psi ,\;(\zeta =0)$$

(22) $$p^{z}=\varphi ,\;(\xi =1)$$

The symbols **φ** and **ψ** denote the specified partial pressures of the individual components resulting from the operating conditions of the membrane.

It should be noticed that iterative correction of permeate-side boundary conditions by means of the Newton method involves solving a system of nonlinear algebraic equations with respect to the desired molar flux densities **N**, i.e.,

(23) F(N)=0

For the support located on the permeate side, i.e., in accordance with Algorithm 1, or on the retentate side, i.e., in accordance with Algorithm 2, the values of the function **F** are calculated, respectively, as

(24) $$F(N)=p^{s}(1)-\psi$$

(25) $$F(N)=p^{z}(1)-\varphi$$

The algorithm for determining the steady states of a specific composite membrane also has two variants, depending on the orientation of the support with respect to the zeolite. They are presented below.

**Algorithm 1** Algorithm for the determination of steady states of composite membranes, for Equations (7) and (8) and boundary conditions (19) and (20)**Step 1:**   Adopt boundary conditions (19) **Step 2:**   Assume preliminary values of molar flux densities **N** (Formula (10)) **Step 3:**   Integrate the system of Equation (7) in the interval ξ ∈ [0, 1] **Step 4:**   Assume **p***^s^*(0) = **p***^z^*(1) **Step 5:**   Integrate the system of Equation (8) **Step 6:**   Check the satisfaction of the boundary condition (20) **Step 7:**   If the boundary condition (20) is not satisfied with the assumed accuracy, then improve the values of **N** according to the external Newton algorithm and return to **Step 3**

**Algorithm 2** Algorithm for the determination of steady states of composite membranes, for Equations (7) and (8) and boundary conditions (21) and (22)**Step 1:**   Adopt boundary conditions (21) **Step 2:**   Assume preliminary values of molar flux densities **N** (Formula (10)) **Step 3:**   Integrate the system of Equation (8) in the interval ζ ∈ [0, 1] **Step 4:**   Assume **p***^z^*(0) = **p***^s^*(1) **Step 5:**   Integrate the system of Equation (7) **Step 6:**   Check the satisfaction of the boundary condition (22) **Step 7:**   If the boundary condition (22) is not satisfied with the assumed accuracy, then improve the values of **N** according to the external Newton algorithm and return to **Step 3**

In Formulas (18)–(25), the vector notation was used, since these relations apply to the general case of *K*-component diffusion. As can be seen, determining the steady state of the composite membrane reduces to solving the boundary value problem, which is achieved in this case using the shooting method. As a result, two groups of information are obtained—that is, the molar flux densities and a set of partial pressure profiles of the individual components in the zeolite and in the support.

The idea presented above for determining steady-state characteristics of composite membranes can be extended to a larger number of layers. In particular, it may include a larger number of supports with varying structural parameters, i.e., thickness, pore diameter, porosity, and tortuosity. The model of a membrane composed of a zeolite film and two support layers is represented by a system including differential Equation (7) and equations describing mass transfer in the supports, i.e.,

(26) $$\frac{dp^{s1}}{d\zeta_{1}}=-L_{s1}RT\cdot B^{p1}\cdot N^{s1}-\frac{B^{e1}}{\eta }\frac{dp_{t1}}{d\zeta_{1}}\cdot E_{1}\cdot p^{s1}$$

(27) $$\frac{dp^{s2}}{d\zeta_{2}}=-L_{s2}RT\cdot B^{p2}\cdot N^{s2}-\frac{B^{e2}}{\eta }\frac{dp_{t2}}{d\zeta_{2}}\cdot E_{2}\cdot p^{s2}$$

where ζ_1_ and ζ_2_ are dimensionless coordinates in the first and second support, respectively. They can be defined as ζ_1_ = *x*_1_/*L_s_*_1_ and ζ_2_ = *x*_2_/*L_s_*_2_. The total thickness of the composite membrane is *L* = *L_z_* + *L_s_*_1_ + *L_s_*_2_.

Since at the steady state there is no mass accumulation, the following equations hold at the inner boundaries of the layers:

(28) $${\left.p^{z}\right|}_{\xi =1}={\left.p^{s1}\right|}_{\zeta_{1}=0}$$

(29) $${\left.p^{s1}\right|}_{\zeta_{1}=1}={\left.p^{s2}\right|}_{\zeta_{2}=0}$$

If the supports are positioned in series on the permeate side, then the boundary conditions on both sides of the membrane, i.e., for ξ = 0 and for ζ_2_ = 1, take the following form:

(30) $$p^{z}=\varphi ,\;(\xi =0)$$

(31) $$p^{s2}=\psi ,\;\left(\zeta_{2}=1\right)$$

**Figure 3:**
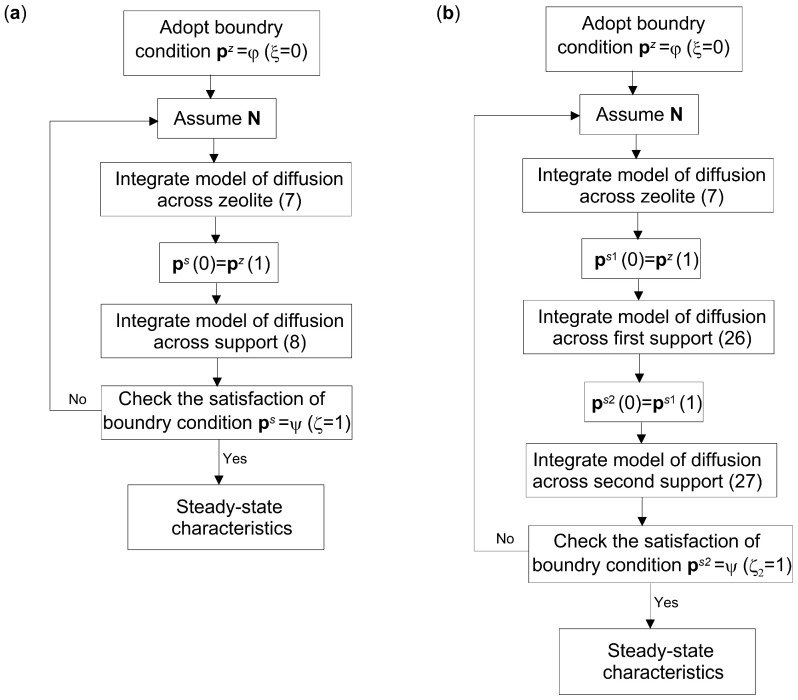
Algorithm for the determination of the steady states of composite membranes for downstream support position: (**a**) membrane with a single support; (**b**) membrane with two supports.

The algorithm for determining the steady states of the membrane with two supports is shown in Figure 3b.

## 3. Results and Discussion

### 3.1. Steady States of the Two-Layer Composite Membrane (Zeolite–Support)

The steady states of composite membranes are realized in industry in continuous flow apparatuses for mixture separation, while in the laboratory, they are realized in a two-sided, open Wicke–Kallenbach diffusion cell.

For a composite membrane consisting of a zeolite film and a support, the feedstock mixture can be supplied on either the zeolite side or the support side. The application of the proposed membrane model and algorithm for determining the steady state of multi-component diffusion for both alternative orientations of the support is illustrated below.

In all numerical experiments, the same thicknesses of the particular layers of the two-layer composite membrane were assumed, i.e., *L_z_* = 4 × 10^−5^ m and *L_s_* = 3 × 10^−3^ m. The thicknesses of the composite membrane layers were selected based on literature reports by several authors [21,33,34,37,39]. This assumption enables comparison of the results for different mixtures and helps to formulate general conclusions. A sufficiently thick support was adopted so that its effect on the operation of the membrane could be observed. The permselective layer consisted of silicalite-1 with porosity ε*_z_* = 0.297 [47] deposited on porous steel support for which ε*_s_*/τ = 0.27 [35].

The steady states were determined for the diffusion of two gas mixtures {A_1_, A_2_}, i.e., {H_2_, CO_2_} and {H_2_, *n*-C_4_H_10_}. The values of the physicochemical parameters that appear in Equations (4)–(6), (7)–(14), and (16) and (17) were adopted based on literature data as provided by Kangas et al. [34] and Krishna and Paschek [21]. The aforementioned authors conducted their research using silicalite-1 as a zeolite film. Krishna and Paschek [21] used a steel porous layer as a support. In contrast, Kangas et al. [34] used a two-layered α-alumina support. The support layers thus differed in both average pore diameter and thickness. In the present study, a single support with a pore diameter of *d_p_* = 5 × 10^−7^ m was used in the numerical computations for all diffusing mixtures. For such pore diameters, mass may be transported by molecular diffusion, Knudsen diffusion, and viscous flow. For this reason, it is reasonable to use the full DGM [48]. The binary diffusion coefficients in the pores of the support were calculated based on the Fuller formula, while the viscosities of the gaseous mixtures were determined using the Wilke formula [49].

The values of physical and chemical parameters used in the calculations are given in Table 1. For pure substances, Langmuir isotherms were employed based on experimental data [27,34]. The adsorption equilibrium of gaseous mixtures was determined based on the Ideal Adsorption Solution (IAS) method [42].

To illustrate the separation effect of the membrane, an equimolar composition of the mixture in the feed stream was assumed, that is, *y*_1_ (0) = *y*_2_ (0) = 0.5. In our analysis for both gas mixtures, pure hydrogen was supplied on the permeate side, i.e., *y*_1_ (*L*) = 1.0. The molar fractions on both sides of the composite membrane chosen in this way help to interpret the results obtained and to formulate useful conclusions concerning the process.

**Figure 4:**
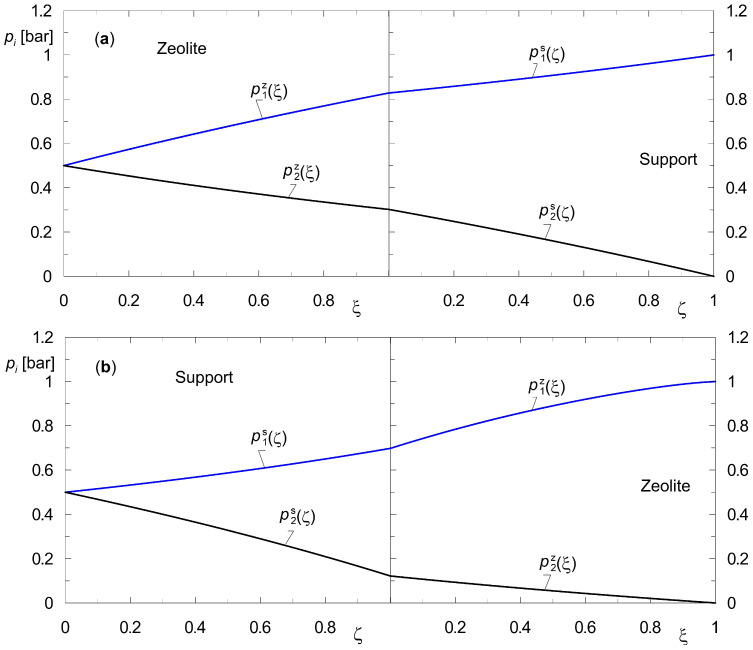
Partial pressure profiles during steady-state binary diffusion of {A_1_, A_2_} = {H_2_, CO_2_} across a composite membrane with a zeolite film with hydrogen as sweep gas; (**a**) downstream support position; (**b**) upstream support position (*p_t_*(0) = *p_t_*(*L*) = 1 bar).

**Figure 5:**
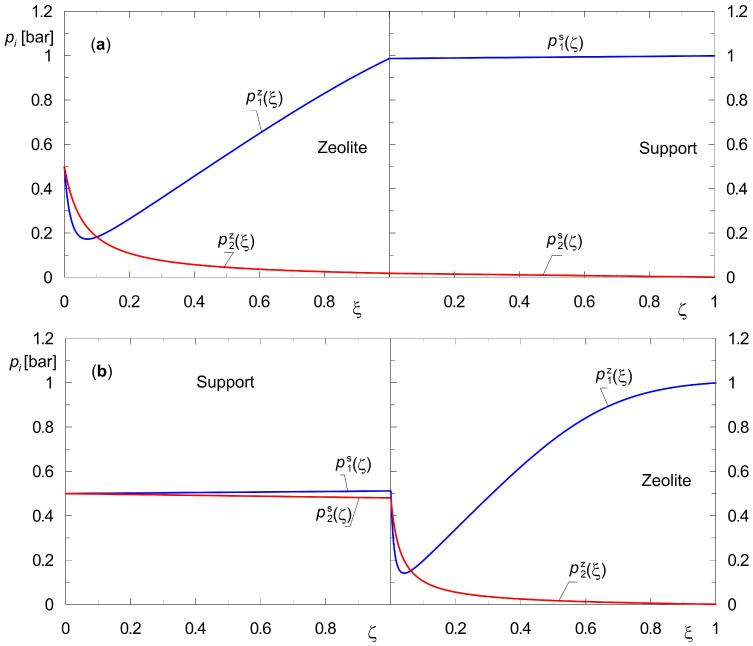
Partial pressure profiles during steady-state binary diffusion of {A_1_, A_2_} = {H_2_, *n*-C_4_H_10_} across a composite membrane with a zeolite film with hydrogen as sweep gas; (**a**) downstream support position; (**b**) upstream support position (*p_t_*(0) = *p_t_*(*L*) = 1 bar).

Figure 4 and Figure 5 show profiles of the state variables, i.e., $p_{i}^{z}\left(\xi \right)$ and $p_{i}^{s}\left(\zeta \right)$ for the diffusion of two different gas mixtures at steady-state conditions. Figure 4 shows profiles corresponding to the diffusion of the mixture {H_2_, CO_2_}, while the profiles $p_{i}^{z}\left(\xi \right)$ and $p_{i}^{s}\left(\zeta \right)$ illustrated in Figure 5 concern the diffusion of {H_2_, *n*-C_4_H_10_}.

Figures labeled with (a) are for the downstream support position, while figures labeled with (b) are for the upstream support position. In the numerical simulations, the same total pressure on both sides of the membrane, *p_t_* = 1 bar, was assumed. This makes it possible to estimate the lower limit of the membrane’s separation capacity, i.e., one that is not influenced by the transmembrane pressure gradient Δ*p_t_*.

Functions $p_{i}^{z}\left(\xi \right)$ and $p_{i}^{s}\left(\zeta \right)$, referred to as steady-state profiles, represent an important group of information about the performance of a specific membrane. It turns out that analyzing these functions yields valuable process information. It also leads to conclusions about how to model the membrane depending on the physicochemical properties of the substances involved in diffusion. Indeed, if the support does not exhibit separation properties, then a given separation process can be modeled using only Equation (7).

A brief review of the results obtained is presented below. It is important to note that the use of partial pressures as state variables in both the support and the zeolite facilitates a pictorial comparison of the separation capabilities for all layers of the composite membrane.

The component A_1_ in the mixture of {A_1_, A_2_} = {H_2_, CO_2_} (Figure 4) adsorbs to a small extent on the surface of silicalite-1 but diffuses easily through the pores of the support. On the other hand, the component A_2_ is a compound that adsorbs more strongly on zeolite. However, it is characterized by lower diffusion coefficients in the pores of the support.

It turns out that if the support is positioned on the retentate side, it already permits a preliminary separation of the analyzed mixtures (Figure 4b). This is due to the different diffusion rates of the components in the pores of the support. The application of the dusty gas model enables the observation of such a phenomenon.

It is important to note that according to Formulas (16) and (17), the diffusion rate in the zeolite is more influenced by the component that adsorbs more strongly, i.e., CO_2_. This is because it is the main one that increases the total coverage θ*_t_* = θ_1_ + θ_2_.

The results of the numerical simulations, as shown in Figure 4, lead to the conclusion that the support affects the partial pressure distributions across the membrane, i.e., both in the zeolite film and in the support. This is a significant conclusion from a technological perspective. In fact, the application and positioning of the support is important not only for the mechanical strength of the membrane, but also for its separation properties. It turns out, however, that it is also relevant to the modeling of membrane separation processes. For gaseous mixtures with moderate adsorption affinities, the presence of the support should be included in the process calculations. In other words, the full Equations (7) and (8) should then be used.

The results of steady-state diffusion simulation for the mixture {A_1_, A_2_} = {H_2_, *n*-C_4_H_10_} across the composite membrane are shown in Figure 5. It is easy to observe qualitative and quantitative differences in the steady-state profiles compared to the results reported in Figure 4. These differences require a process explanation, which is provided below.

**Figure 6:**
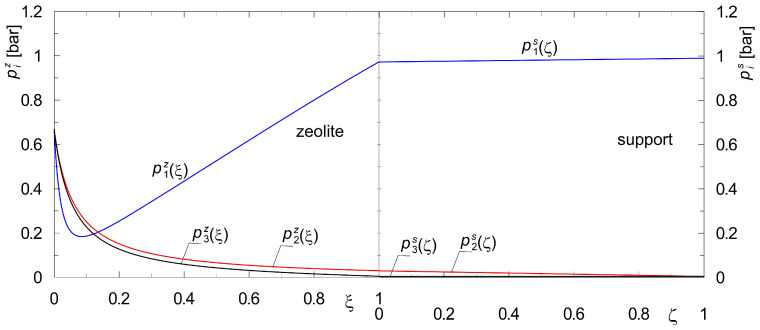
Partial pressure profiles during steady-state diffusion of {A_1_, A_2_, A_3_} = {H_2_, *n*-C_4_H_10_, CO_2_} across a composite membrane with a zeolite film with lack of carbon dioxide at permeate side (*q*_1_^*^ = 0.7323 mol·kg^−1^, *q*_2_^*^ = 2.1953 mol·kg^−1^, *q*_3_^*^ = 2.025 mol·kg^−1^; *b*_1_ = 1.1089 × 10^−5^ Pa^−1^, *b*_2_ = 1.1 × 10^−3^ Pa^−1^, *b*_3_ = 1.8377 × 10^−5^ Pa^−1^).

Butane belongs to the strongly adsorbing compounds. As shown in an earlier publication [41,43], the effect of *n*-butane adsorption on inhibiting surface diffusion in zeolites is enormous. Moreover, due to the size of its molecules, it diffuses much more slowly through the pores of the support compared to hydrogen. These two elements, which influence the rate of diffusion in both layers of the membrane, yield the partial pressure distributions shown in Figure 5. Let us consider Figure 5a. The separation of the mixture takes place practically in the zeolite film. In contrast, Figure 5b shows that neither the presence of the support nor its location affects the final result of the separation of the components. This means that the steady-state model (Equations (7) and (8)) may be reduced to Equation (7). Similar conclusions can be drawn based on the diffusion of a three-component mixture {H_2_, *n*-C_4_H_10_, CO_2_} across a composite membrane. Such results are presented in Figure 6. We hypothesize that the observed phenomenon will also be present during the diffusion of a multi-component mixture, where only one component exhibits a significantly higher adsorption affinity compared to the other components of the mixture.

In our opinion, the primary reason for the strong curvilinearity of the partial pressure profiles of the two components within the zeolite film is the high adsorption affinity of *n*-butane. A significant increase in the total coverage leads to a lowering of the surface diffusion coefficients in the zeolite, in accordance with Equations (15)–(17).

### 3.2. Steady State of a Three-Layer Composite Membrane (Zeolite–Support 1–Support 2)

**Figure 7:**
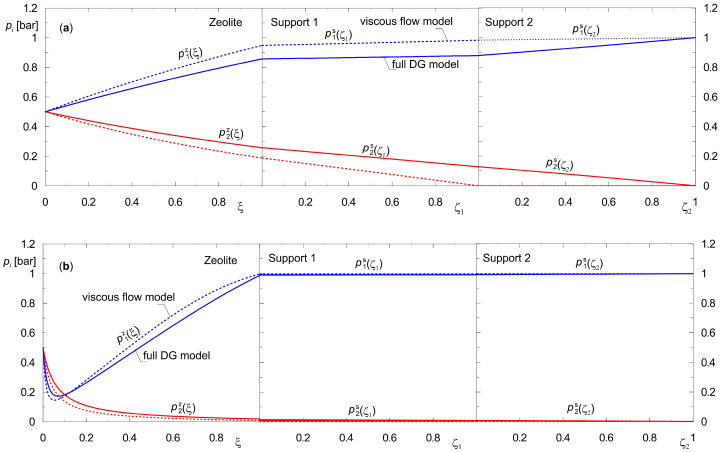
Partial pressure profiles during steady-state binary diffusion of {A_1_, A_2_} across a three-layer composite membrane with hydrogen as sweep gas; (**a**) {H_2_, CO_2_} (**b**) {H_2_, *n*-C_4_H_10_} (downstream support position) *p*_0_(0) = *p_t_*_2_(1) = 1 bar.

The partial pressure profiles of the components of the mixtures {H_2_, CO_2_} and {H_2_, *n*-C_4_H_10_} in a composite membrane composed of a zeolite film and two layers of supports characterized by different structural properties are shown in Figure 7. Silicalite-1, with a thickness of *L_z_* = 4 × 10^−5^ m, was adopted as the zeolite in all calculations. The geometrical characteristics of the supports are shown in Table 2. The selection of parameters for the supports was based on literature data [33,34,50]. The separation of an equimolar mixture with a total pressure of 1 bar was simulated with pure hydrogen at 1 bar on the permeate side.

According to Jareman et al. [51], Wirawan et al. [33], and Kangas et al. [34], in the second layer of the support, characterized as outlined in Table 2, mass transport occurs solely through viscous flow. This layer, therefore, does not exhibit separation properties. However, according to the authors, a full dusty gas model should be used for both support layers. A comparison of the membrane’s performance according to these two approaches for describing the mass transport is shown in Figure 7. The solid lines indicate the partial pressure profiles calculated from the full DG model in both support layers. In contrast, the dashed lines refer to the consideration of solely the viscous flow model in the second support.

The partial pressure distributions of the components of the mixture {H_2,_ CO_2_} (Figure 7a) demonstrate the importance of including supports in the modeling of composite membranes for the separation of substances with moderate adsorption strength. There is a significant difference in the results when considering only the viscous flow for the second macroporous support compared to the full dusty gas model. Moreover, the way the supports are modeled also influences the concentration distributions in the zeolite.

While analyzing the results obtained for the mixture {H_2_, *n*-C_4_H_10_} (Figure 7b), it can be concluded that its separation occurs only in the zeolite. This means that when formulating a mathematical model of diffusion in a composite membrane, one can limit only to equations describing mass transport in the zeolite. This is consistent with the results obtained for a composite membrane with a single support (Figure 5 and Figure 6).

The second group of information concerning the performance of a particular membrane is a set of molar flux densities *N_i_* of the individual components. These are important measurable quantities that permit the calculation of the yield and selectivity of the membrane.

**Figure 8:**
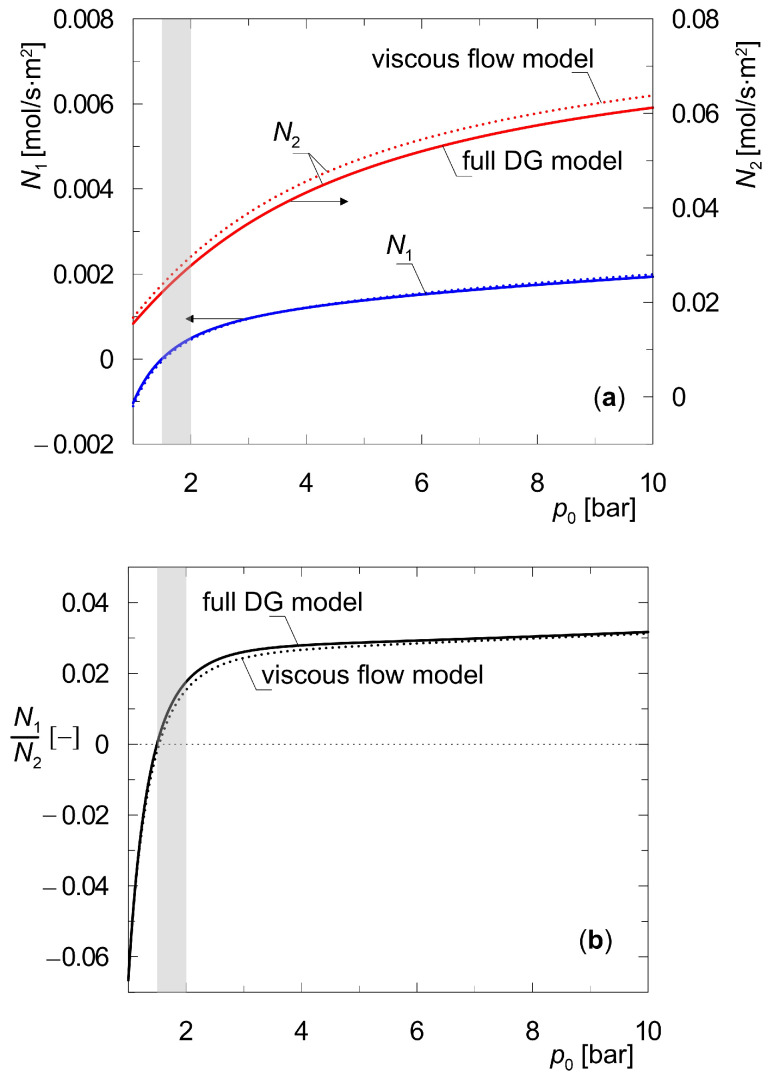
Effect of transmembrane pressure gradient on molar flux densities *N_i_* during steady-state diffusion of a binary mixture {H_2_, CO_2_} through a three-layer composite membrane; (**a**) the molar flux densities; (**b**) the ratio of molar flux desities (downstream support position) *p_t_*_2_(1) = 1 bar; *y*_1_(0) = *y*_2_(0) = 0.5; *y*_1_(*L*) ≅ 1.0.

The profiles of partial pressures shown above in Figure 4, Figure 5, Figure 6 and Figure 7 correspond to conditions in which the total pressure is equal on both sides of the composite membrane. The proposed method for determining steady states can also be used to determine and explain the effect of the transmembrane total pressure gradient Δ*p_t_* on molar flux densities. In line with common opinion, increasing the transmembrane pressure gradient is expected to intensify mass transport. Figure 8 shows the effect of raising the transmembrane pressure gradient on molecular flux densities *N_i_* for a mixture of {H_2_, CO_2_}. The total pressure on the permeate side is maintained at 1 bar, while the retentate pressure is varied. Calculations were carried out for moderate feed pressures *p*_0_, which are easily implemented in laboratory practice [35].

As can be seen in Figure 8, the molar flux densities of both components, *N_i_*, increase throughout the entire range of transmembrane pressure gradient shown, albeit to varying degrees. The rise in molar flux density of component A_2_, which has a higher adsorption affinity, is more pronounced. In spite of the evident quantitative and qualitative differences between the partial pressure profiles in Figure 7a obtained from the viscous flow model and full DG model, the differences in molar flux density values are negligible. They mainly concern the heavier component, i.e., CO_2_, which features lower pore diffusion coefficients than component A_1_. The viscous flow is non-selective with respect to the mixture components. Thus, the molar flux densities of *N*_2_ determined from the viscous flow model only are higher than those obtained from the full DG model.

Figure 8 shows the results of simulations in which there is an imposition of mass transport due to the partial pressure gradient of hydrogen (A_1_) and the total pressure difference on both sides of the membrane. It was observed that there exists a certain pressure range *p*_0_ ∈ [1.5–2.0] bar for which there is a negative partial pressure gradient of the component A_1_, but a positive total pressure gradient (*p*_0_ > *p_t_*). In that case, hydrogen moves from its lower partial pressure to a higher one. This range is marked in gray in Figure 8.

**Figure 9:**
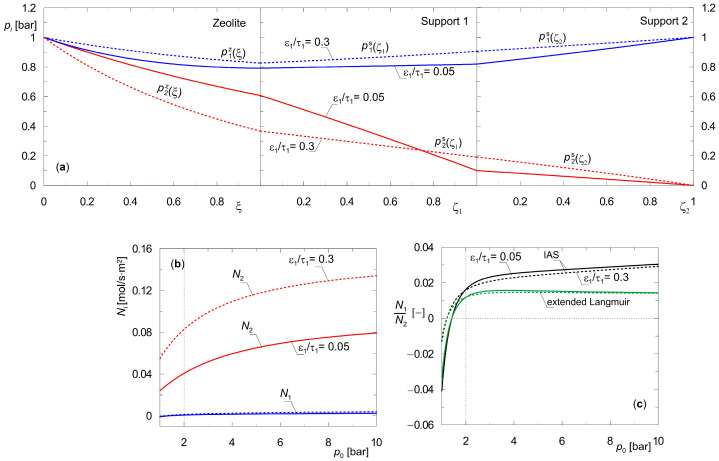
Steady-state characteristics of binary diffusion of {H_2_, CO_2_} across a three-layer composite membrane with different structural parameters of first support layer; (**a**) partial pressure profiles *p*_0_(0) = *p_t_*(*L*) = 2 bar; (**b**,**c**) effect of transmembrane pressure gradient on molar flux densities *N_i_* for *p_t_*(*L*) = 1 bar (downstream support position), *y*_1_(0) = *y*_2_(0) = 0.5; *y*_1_(*L*) ≅ 1.0.

Additional simulations were conducted to investigate the impact of the key structural property of the supports, which, in addition to pore diameter, is the ratio of porosity to channel tortuosity (ε_s_/τ). Figure 9 shows the results. Partial pressure profiles were obtained using a total pressure difference of Δ*p_t_* = 1 bar. In that case, the values of partial pressure of the component A_1_, i.e., hydrogen, are equal on both sides of the composite membrane (Figure 9a). The analysis was carried out by varying the parameter (ε_s_/τ)_1_, i.e., the support adjacent to the zeolite layer, while keeping the other parameters as listed in Table 2.

The first support provides additional separation of the components of the diffusing mixture due to the significant contribution of Knudsen diffusion to the resultant mass transfer rate. This is due to the small pore diameters of this layer *d_p_*_1_. This phenomenon is confirmed by the shape and location of the partial pressure profiles, as well as by the molar flux densities. The use of a support with a larger ratio (ε_s_/τ)_1_ leads to a smaller contribution of this layer to the separation of a given gas mixture (Figure 9a) and higher molar flux densities (Figure 9b). Thus, the structural parameters of the support affect the separation capacity of the composite membrane as a whole.

In addition, Figure 9c shows the ratio of molar flux densities of components A_1_ and A_2_ obtained using extended Langmuir isotherms, i.e., a model simplified with respect to IAST. Green lines, solid and dashed, indicate these results. In the range of moderate total pressures *p*_0_, the steady-state branches obtained from the IAS model and extended Langmuir isotherms overlap. As the feed pressure *p*_0_ increases, the *N*_1_/*N*_2_ values obtained according to these two methods tend to differ more and more. This is consistent with the thermodynamic interpretation of interphase equilibrium and is due to the different values of the saturation concentrations of the two components.

## 4. Conclusions

A critical comparison and evaluation of the methods for the determination of steady states in composite membranes encountered in the literature does not provide a clear answer as to the correctness and accuracy of the determination of these states. Our own studies of the dynamics yielded a similar opinion. Taking these into account, a universal, clear, and accurate method for determining the steady states of composite membranes in which multi-component diffusion of gaseous mixtures occurs was proposed. A well-known shooting algorithm was employed to solve nonlinear boundary value problems. The method can be used for simulation and process design of membranes with any support orientation with respect to the zeolite film.

The presented algorithm was implemented in this work to study the influence of the adsorption affinity of the components in the diffusion mixture and the location of the support on the aforementioned steady-state characteristics. For this reason, two gas mixtures, i.e., {H_2_, CO_2_} and {H_2_, *n*-C_4_H_10_}, were selected for numerical simulations. In each of these binary mixtures, there is a weakly adsorbable component A_1_ and a more strongly adsorbable component A_2_.

Based on the dynamic analysis of zeolite layers (Figure 2), it was demonstrated that the reasoning of arriving at a steady state is not an unambiguous issue and depends on the choice of state variable. Thus, if dynamic simulations determine the steady state, then the time trajectories of all state variables must be tracked. To evaluate the arrival at the steady state, it is necessary to choose the quantity that changes most slowly and to provide a criterion for achieving this state. It was demonstrated that utilizing models where the state variables are the partial pressures of diffusing components is advantageous. This is because they tend toward the steady state in the slowest way.

A more straightforward and unambiguous way to determine the steady states of microporous membranes is to use models that describe steady-state conditions. This is because they do not require providing a criterion for achieving these states. Such a method was applied in this work. The concept of the proposed method can be applied to model membranes with any number of both selective layers and supports.

Based on partial pressure profiles in the zeolite film and the support, the separation performance of the two configurations of the considered composite membranes was compared. The separation performance of these membranes was correlated with the adsorption affinity of the mixture components. The stronger the component adsorbs on the zeolite surface, the smaller the contribution of the support in the separation becomes. This is confirmed by the partial pressure profiles (Figure 4 and Figure 5). For mixture {H_2_, CO_2_}, there was a change in the partial pressure of component A_2_ by about 60% in the support alone, while for mixture {H_2_, *n*-C_4_H_10_}, it was less than 2%. Thus, for mixtures in which one of the components is expected to be strongly adsorbable, the diffusion resistance of the support can be neglected. The steady-state mathematical model is then simplified to equations describing surface diffusion in the zeolite film.

The effect of varying the structural parameters of the supports on the performance of three-layer membranes was discussed in detail. It was demonstrated that it is reasonable to use full DGM to describe mass transport even in wide-porous supports. This is because the way the supports are modeled affects not only the distribution of concentrations in the supports themselves, but also the performance of the zeolite layer.

Based on the results discussed above, it may be concluded that a comprehensive understanding of the steady-state characteristics of composite membranes requires the simultaneous analysis and interpretation of both concentration profiles, expressed here by partial pressures, as well as molar flux densities of *N_i_*. Experimental studies alone are insufficient for selecting a specific membrane and performing process calculations. Empirical determination of membrane partial pressure profiles is essentially infeasible or marginally difficult to realize. Therefore, numerical simulations are a valuable complementary component of experimental studies. In the authors’ opinion, the developed algorithms for determining the steady-state conditions of composite membranes should be a valuable and standard tool for researchers and engineers.

The presented model can be applied to any composite membrane (i.e., with any type and number of layers) and any number of diffusing components. This feature results from the fact that the model parameters can be adjusted depending on the type of membrane and the composition of the mixture that is subject to separation. It is possible to modify the method by including the presence of non-adsorbable inert gases. At higher pressures, the activity coefficients of gases differ significantly from unity. In this case, only the part of the algorithm concerning phase equilibrium in zeolite changes, and the RAS theory should be used instead of the IAS theory. The proposed algorithm is expected to be a tool for verifying composite membrane dynamics studies. Apart from its cognitive aspects, the work is addressed to practicing engineers involved in the design of membrane processes, i.e., the selection of membranes, the number of layers, and the rational selection of operating conditions for such facilities.

## Figures and Tables

**Table 1:** Values of basic physicochemical parameters adopted in numerical tests [21,27,34].

Parameter	{A_1_, A_2_} = {H_2_, CO_2_}	{A_1_, A_2_} = {H_2_, *n*-C_4_H_10_}
$q_{1}^{*}$ [mol·kg^−1^]	5.4	0.7323
$q_{2}^{*}$ [mol·kg^−1^]	2.025	2.1953
ρ*_z_* [kg·m^−3^]	2503	2560
$D_{1}^{z}\left(0\right)$ [m^2^·s^−1^]	7.4153 × 10^−8^	1.0 × 10^−9^
$D_{2}^{z}\left(0\right)$ [m^2^·s^−1^]	3.81 × 10^−9^	5.0 × 10^−11^
*b*_1_ [Pa^−1^]	4.703 × 10^−8^	1.1089 × 10^−5^
*b*_2_ [Pa^−1^]	1.8377 × 10^−5^	1.1 × 10^−3^
References	Kangas et al. (2013) [34]	Kapteijn et al. (1995) [27] Krishna and Paschek (2000) [21]

**Table 2:** Geometrical and structural parameters of support layers (*i* = 1, 2).

Parameter	Support 1	Support 2
*L_si_* [m]	3.0 × 10^−5^	3.0 × 10^−3^
*d_pi_* [m]	1.0 × 10^−7^	3.0 × 10^−6^
(ε/τ)*_i_*	0.027	0.50

## Data Availability

The original contributions presented in this study are included in the article. Further inquiries can be directed to the corresponding author.

## Nomenclature and Abbreviations

A*_i_*	The *i*-th component.
{A_1_, A_2_}	Mixture with composition A_1_, A_2_.
*B^e^*	Effective permeability coefficient, m^2^.
*b_i_*	Langmuir parameter, Pa^−1^, bar^−1^.
*d_p_*	Pore diameter in the support, m.
$D_{ij}^{e}$	Effective binary Maxwell–Stefan diffusion coefficient, m^2^·s^−1^.
$D_{i}^{z}$	Maxwell–Stefan diffusion coefficient in zeolite, m^2^·s^−1^.
$D_{ij}^{z}$	Binary Maxwell–Stefan diffusion coefficients in zeolite, m^2^·s^−1^.
$D_{Kn,i}^{e}$	Effective Knudsen diffusion coefficient of the *i*-th component, m^2^·s^−1^.
DGM	Dusty gas model.
*F_V_*	Volumetric flow rate, m^3^·s^−1^.
IAS	Ideal adsorption solution.
**J**	Jacobi matrix d**q**/d**p**.
*K*	Number of species.
*K*_0_	Structural parameter of the porous medium, m.
*L_s_*, *L_z_*	Thickness of the support and zeolite film, respectively, m.
*L*	Total thickness of the composite membrane, m.
*M_i_*	Molar mass of species *i,* kg·mol^−1^.
MS	Maxwell–Stefan model.
*N_i_*	Molar flux density of the *i*-th component, mol·s^−1^·m^−2^.
**N**	Vector of molar flux densities, mol·s^−1^·m^−2^.
*p_t_*	Total pressure, bar, Pa, in figures.
Δ*p_t_*	Transmembrane gradient of total pressure, bar.
*p_i_*	Partial pressure of *i*-th component, bar or Pa.
**p**	Vector of partial pressures, bar or Pa.
*q_i_*	Molar concentration in the adsorbed phase, mol·kg^−1^.
$q_{i}^{*}$	Saturation concentration, mol·kg^−1^.
**q**	Vector of concentrations in the adsorbed phase, mol·kg^−1^.
*R*	Universal gas constant, J·mol^−1^·K^−1^.
*t*	Time, s.
*T*	Temperature, K.
*V*	Volume of chamber of the W-K diffusion cell, m^3^.
W-K	Wicke–Kallenbach.
*x*	Spatial coordinate in support, m.
*y_i_*	Molar fraction of *i*-th component in gas phase.
*z*	Spatial coordinate in zeolite, m.
Greek letters	
Γ*_ij_*	Thermodynamic factors.
**Γ**	Matrix of thermodynamic factors.
ε*_s_*, ε*_z_*	Support and zeolite porosity, respectively.
θ*_i_*	Fractional occupancy of *i*-th component.
θ*_t_*	Total surface coverage.
**θ**	Vector of fractional surface occupancies.
μ*_i_*	Chemical potential, J·mol^−1^.
ξ	Dimensionless spatial coordinate in zeolite film.
ζ	Dimensionless spatial coordinate in support.
η	Viscosity of the gaseous mixture, Pa·s.
ρ*_z_*	Solid density of zeolite, kg·m^−3^.
τ	Tortuosity factor.
Superscripts	
^O^	Refers to the standard state.
*g*	Refers to gas phase.
*z*	Refers to zeolite surface or diffusion in zeolite.
*s*	Refers to diffusion in support.
*si*	*i*-th support in three-layer composite membrane.
Subscripts	
0	Refers to feed side of composite membrane.
*f*	Feed stream.
*i*,*j*	*i*-th/*j*-th species in mixture.
*L*	Refers to permeate side of composite membrane.
